# Stability of Neuronal Networks with Homeostatic Regulation

**DOI:** 10.1371/journal.pcbi.1004357

**Published:** 2015-07-08

**Authors:** Daniel Harnack, Miha Pelko, Antoine Chaillet, Yacine Chitour, Mark C.W. van Rossum

**Affiliations:** 1 School of Informatics, University of Edinburgh, Edinburgh, United Kingdom; 2 L2S—Univ. Paris Sud—CentraleSupélec, Gif sur Yvette, France; École Normale Supérieure, College de France, CNRS, FRANCE

## Abstract

Neurons are equipped with homeostatic mechanisms that counteract long-term perturbations of their average activity and thereby keep neurons in a healthy and information-rich operating regime. While homeostasis is believed to be crucial for neural function, a systematic analysis of homeostatic control has largely been lacking. The analysis presented here analyses the necessary conditions for stable homeostatic control. We consider networks of neurons with homeostasis and show that homeostatic control that is stable for single neurons, can destabilize activity in otherwise stable recurrent networks leading to strong non-abating oscillations in the activity. This instability can be prevented by slowing down the homeostatic control. The stronger the network recurrence, the slower the homeostasis has to be. Next, we consider how non-linearities in the neural activation function affect these constraints. Finally, we consider the case that homeostatic feedback is mediated via a cascade of multiple intermediate stages. Counter-intuitively, the addition of extra stages in the homeostatic control loop further destabilizes activity in single neurons and networks. Our theoretical framework for homeostasis thus reveals previously unconsidered constraints on homeostasis in biological networks, and identifies conditions that require the slow time-constants of homeostatic regulation observed experimentally.

## Author Summary

Despite their apparent robustness many biological system work best in controlled environments, the tightly regulated mammalian body temperature being a good example. Biological homeostatic control systems, not unlike those used in engineering, ensure that the right operating conditions are met. Similarly, neurons appear to adjust the amount of activity they produce to be neither too high nor too low by, among other ways, regulating their excitability. However, for no apparent reason the neural homeostatic processes are very slow, taking hours or even days to regulate the neuron. Here we use results from mathematical control theory to examine under which conditions such slow control is necessary to prevent instabilities that lead to strong, sustained oscillations in the activity. Our results lead to a deeper understanding of neural homeostasis and can help the design of artificial neural systems.

## Introduction

Neurons in the brain are subject to varying conditions. Developmental processes, synaptic plasticity, changes in the sensory signals, and tissue damage can lead to under- or overstimulation of neurons. Both under- and overstimulation are undesirable: prolonged periods of excessive activity are potentially damaging and energy inefficient, while prolonged low activity is information poor. Neural homeostasis is believed to prevent these situations by adjusting the neural parameters and keeping neurons in an optimal operating regime. Such a regime can be defined from information processing requirements [1, 2], possibly supplemented with constraints on energy consumption [3]. As homeostasis can greatly enhance computational power [4–6], and a number of diseases have been linked to deficits in homeostasis [7–10], it is important to know the fundamental properties of homeostatic regulation, its failure modes, and its constraints.

One distinguishes two homeostatic mechanisms: synaptic and intrinsic excitability homeostasis [11, 12]. In case of over-excitement, synaptic homeostasis scales excitatory synapses down and inhibitory synapses up, while intrinsic homeostasis increases the firing threshold of neurons. Intrinsic homeostasis is the subject of this study. Intrinsic homeostasis correlates biophysically to changes in the density of voltage gated ion channels, [13–16], as well as the ion channel location in the axon hillock [17, 18].

All homeostatic mechanisms include an activity sensor and a negative feedback that counters deviations of the activity from a desired value. Control theory describes the properties of feedback controllers and the role of its parameters [19]. In engineering one typically strives to bring a system rapidly to its desired state with minimal residual error. It is reasonable to assume that neural homeostasis has to be fairly rapid too in order to be effective, although it should not interfere with the typical timescales of perceptual input or of neural processing (millisecond to seconds). However, intrinsic excitability homeostasis is typically much slower, on the order of many hours to days ([13, 15, 20, 21], but see [14]). One hypothesis is that this is sufficiently fast to keep up with typical natural perturbations, but an alternative hypothesis, explored here, is that stable control necessitates such slow homeostasis. Note that the speed of homeostasis is the time it takes to reach a new equilibrium after a perturbation and does not rule out that homeostatic compensation can start immediately after the perturbation; it just takes a long time to reach its final value.

In computational studies homeostatic parameters are usually adjusted by hand to prevent instability, but a systematic treatment, in particular in networks, is lacking. In a recent study a network with excitatory and inhibitory populations with distinct homeostatic control was studied and with linear stability analysis it was found that instabilities can occur when the inhibitory homeostasis is faster than the excitatory one [22]. However, numerous questions remain: Is homeostatic control consisting of multiple stages equally stable? How do non-linearities in the input-output relation of the neurons affect results? Finally, because in that study a separation of time-scales between the neural activity and homeostasis was assumed, only a constraint on the ratio of homeostatic speeds of the two populations was found, but not on their absolute speeds. It raises the question how the homeostatic speed relates to the neural time-constants.

In this study we analyze three aspects of the stability conditions for networks of neurons equipped with homeostasis. 1) We show that homeostasis can destabilize otherwise stable networks and that, depending on the amount of recurrence, stable homeostatic feedback needs to be slower for networks than for single neurons. 2) We analyze how homeostatic stability is affected by non-linearities in the neuron’s input-output relation. In general systems with non-linearities require slower homeostasis than linear analysis predicts. 3) We show that having multiple intermediate stages in the feedback loop, common in biological signaling cascades, tends to destabilize control, despite the overall feedback being slower. The results put constraints on the design and interpretation of homeostatic control and help to understand biological homeostasis.

## Results

### Homeostatic framework

**Fig 1:**
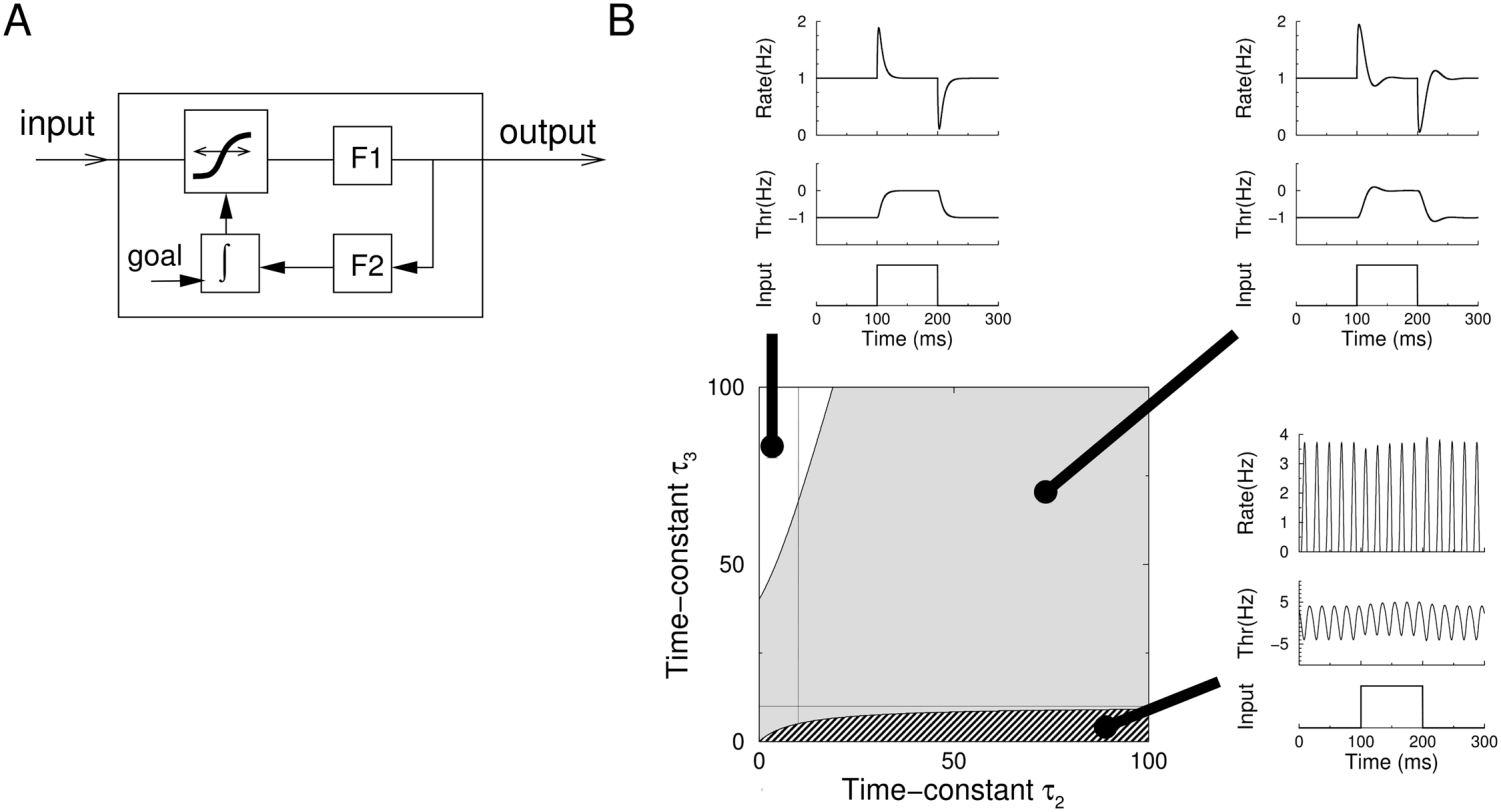
Single neuron homeostasis. A) Schematic illustration of the homeostatic model. The input current is transformed through an input-output relation and a filter. The input-output curve is shifted by a filtered and integrated copy of the output firing rate, so that the average activity matches a preset goal value. *F1* (time-constant *τ*_1_) denotes a filter describing the filtering between input and output of the neuron; *F2* (time-constant *τ*_2_) is a filter between the output and the homeostatic controller. B) The response of the model for various settings of the homeostatic time-constants. The value of *τ*_1_ was fixed to 10ms (thin lines), while *τ*_2_ and *τ*_3_ were varied. Center plot: the response of the neuron can either be stable (top left plot; white region), a damped oscillation (top right plot, gray region), or unstable (bottom right plot, striped region). The surrounding plots show the firing rate of the neuron and the threshold setting in response to a step stimulus.

To examine the stability of homeostatic control we first analyze a single neuron with homeostasis, a schematic is shown in Fig 1A. We describe the activity of the neuron as a function of time with a firing rate *r*_1_(*t*). A common approximation for the firing rate dynamics is

$$\begin{array}{c} \tau_{1}\frac{dr_{1}\left(t\right)}{dt}=-r_{1}\left(t\right)+g\left(u\left(t\right)-\theta \left(t\right)\right) \end{array}$$ (1)

which can be understood as follows: The time-constant *τ*_1_ determines how rapidly the firing rate changes in response to changes in the input and how rapidly it decays in the absence of input. We use *τ*_1_ = 10 ms. The value of *τ*_1_ serves as the time-constant with respect to which all the other time-constants in the system will be defined. As only the ratios between time-constants will matter, the results are straightforwardly adapted to other values of *τ*_1_.

The f-I curve *g*() describes the relation between net input to the neuron and its firing rate. We assume that homeostasis acts effectively as a bias current which shifts the f-I curve, consistent with experimental data [15]. The total input is *u*(*t*) − *θ*(*t*), where *u*(*t*) is proportional to external input current to the neuron, typically from synaptic input. Crucially, *θ*(*t*) is the homeostatically controlled firing threshold of the neuron. While physiologically both the threshold current and threshold voltage of neurons are affected by homeostasis [13], our model comprises both indistinguishably.

The homeostatic controller takes its input from averaged activity, rather than reading out activity directly (see also section Cascaded Homeostatic Control below). To obtain the averaged activity *r*_2_(*t*) of the neuron, the firing rate *r*_1_(*t*) is filtered with a linear first order filter with a time-constant *τ*_2_

$$\begin{array}{c} \tau_{2}\frac{dr_{2}\left(t\right)}{dt}=-r_{2}\left(t\right)+r_{1}\left(t\right) \end{array}$$ (2)

Biophysically, the intra-cellular calcium concentration is a very likely candidate for this sensor [11] in which case *τ*_2_ is around 50ms.

The last step in the model is to integrate the difference between the average activity and the pre-defined desired activity level *r*_*goal*_

$$\begin{array}{c} \tau_{3}\frac{dr_{3}\left(t\right)}{dt}=r_{2}\left(t\right)-r_{goal} \end{array}$$ (3)

*r*_*goal*_ was typically set to 1Hz, but its value is inconsequential. The feedback loop is closed by setting the threshold in Eq (1) equal to this signal, that is *θ*(*t*) = *r*_3_(*t*). Thus, if the activity remains high for too long, *r*_2_ and *r*_3_ increase, increasing the threshold and lowering the firing rate, and vice versa if the activity is below the set-point *r*_*goal*_ for too long.

An attentive reader might have noticed that *θ* is a current, while *r*_3_ is a rate. Formally this inconsistency can be resolved by defining *θ*(*t*) = *γr*_3_(*t*) where *γ* has dimensions A/Hz, and by giving *α* (defined below) dimensions Hz/A. However, for simplicity we use dimensionless units; this does in no way affect our results. Also note, that while *r*_1_ is assumed positive, *r*_3_ and *θ* are not as they are the difference between actual and goal rate and thus can take negative values.

Note that in contrast to the earlier equations, Eq (3) does not have a decay term on the right hand side, i.e. a term of the form −*r*_3_(*t*). This means that instead of a leaky integrator, it is a perfect integrator which keeps accumulating the error in the rate (*r*_2_(*t*) − *r*_*goal*_) without any decay. Mathematically, this can be seen by re-writing Eq (3) as $r_{3}(t)=\frac{1}{\tau_{3}}\int_{-\infty }^{t}[r_{2}(t^{\prime })-r_{goal}]\;dt^{\prime }$. Perfect integrators are commonly used in engineering solutions such as PID controllers and are very robust. A perfect integrator ensures that, provided the system is stable, the goal value *r*_*goal*_ is eventually always reached, as otherwise *r*_3_(*t*) keeps accumulating. The time-constant *τ*_3_ is therefore not strictly a filter time-constant, but it determines how rapidly errors are integrated and thus how quickly homeostasis acts. Although it might appear challenging to build perfect integrators in biology, evidence for them has been found in bacterial chemotaxis [23, 24]. It is straightforward to extend our theory to a leaky integrator; for small leaks, this does not affect our results.

### Local stability of homeostatic control in the single neuron

In general the f-I curve is non-linear. To determine the stability to small perturbations around the homeostatic set-point, a linear approximation of the f-I curve is made, *g*(*x* ^⋆^) = *α*(*x* − *x* ^⋆^), where *x* ^⋆^ = *g* ^−1^(*r*_*goal*_) is the total input at the set-point, and *α* is the slope of the f/I curve at the set-point. An extension to general non-linear f-I curves is presented below.

Using the linearization, we can borrow results from linear control theory to examine the stability of the set of differential equations that define the neural and homeostatic dynamics. One needs to solve the differential equations at the equilibrium point and check whether the solutions diverge. Various equivalent approaches have been developed to determine stability of controllers [25]. Here we write the set of first order equations, Eqs (1)–(3) in matrix form

$$\begin{array}{c} \frac{d}{dt}(\begin{array}{c} r_{1}\left(t\right)\\ r_{2}\left(t\right)\\ r_{3}\left(t\right) \end{array})=M(\begin{array}{c} r_{1}\left(t\right)\\ r_{2}\left(t\right)\\ r_{3}\left(t\right) \end{array})+b\left(t\right) \end{array}$$

with matrix

$$\begin{array}{c} M=(\begin{array}{ccc} -\frac{1}{\tau_{1}} & 0 & -\frac{\alpha }{\tau_{1}}\\ \frac{1}{\tau_{2}} & -\frac{1}{\tau_{2}} & 0\\ 0 & \frac{1}{\tau_{3}} & 0 \end{array}) \end{array}$$

and vector

$$\begin{array}{c} b\left(t\right)=(\begin{array}{c} \begin{array}{c} \frac{\alpha }{\tau_{1}}u\left(t\right)\\ 0 \end{array}\\ -\frac{1}{\tau_{3}}r_{goal} \end{array}) \end{array}$$

In this linearized case, the gain *α* can be absorbed in *τ*_3_. A shallower f-I curve (*α* < 1) implies a weaker feedback, and is fully equivalent to a proportionally slower *τ*_3_; in both cases it takes longer for the system to attain the goal value. In the limit that *τ*_3_ ≫ *τ*_1_, *τ*_2_ the firing rate settles exponentially with a time-constant *τ*_3_/*α* in response to a perturbation, that is, $r_{1}(t)-r_{goal}\propto {\text{e}}^{-\alpha t/\tau_{3}}$.

The theory of differential equations states that the solution to the set of equations is the sum of a particular solution (which is unimportant for our purposes) and solutions to the homogeneous equation, which is the equation with **b** = **0**. With the ‘ansatz’ *r*_*i*_(*t*) = *c*_*i*_ *e* ^*λt*^, one finds that in order to solve the homogeneous equation, the vector **c** = (*c*_1,_ *c*_2_, *c*_3_) must be an eigenvector of *M* with eigenvalue *λ*. This means that *λ* has to solve the characteristic polynomial, det(*M* − *λI*) = −(1 + *τ*_1_ *λ*)(1 + *τ*_2_ *λ*)*τ*_3_ *λ* − *α* = 0. The three eigenvalues of *M* are in general complex numbers and determine the stability of each mode as follows:

- If an eigenvalue is real and negative, the corresponding mode is stable as the exponential e^*λt*^ decays to zero over time.
- If an eigenvalue is complex and the real part is negative, the corresponding mode decays over time as a damped oscillation. In the context of homeostasis such activity oscillations might be biologically undesirable, in particular when they persist for many cycles.
- Finally, the solution of the linearized system will diverge if any of the eigenvalues has a positive real part. In practice, some mechanism, such as a squashing or rectifying f-I curve, will restrain the firing rate and strong sustained oscillations in the firing rate will occur. (For two dimensional systems this can be proven using the Poincare-Bendixson theorem [26]). In this case homeostatic control is unstable.

Which of these above scenarios occurs depends in our model solely on the ratio between the three *τ*_*i*_ parameters. In most of what follows, we determine the required value of *τ*_3_ for given *τ*_1_ and *τ*_2_, i.e. we determine the required homeostatic timeconstant given the neural and calcium timeconstants.

Fig 1B shows simulated responses of a single neuron’s firing rate *r*_1_(*t*), and the threshold variable *r*_3_(*t*) to a step input for various settings of the time-constants. It can be observed that only for extremely short values of *τ*_3_ the neuron is unstable (striped region). In this case the firing rate oscillates continuously. In the gray region the neuron is stable but displays damped oscillations after changes in the activity. Stability without oscillation (white region) can always be achieved by taking *τ*_3_ slow enough. The explicit stability condition follows from the Routh–Hurwitz stability criterion (see Methods). It yields

$$\begin{array}{c} \tau_{3}>\tau_{3}^{0} \end{array}$$

where

$$\begin{array}{c} \tau_{3}^{0}\equiv \alpha \frac{\tau_{1}\tau_{2}}{\tau_{1}+\tau_{2}} \end{array}$$

The above stability criterion confirms the intuition that slower feedback is more stable than fast feedback. When *τ*_2_ is 50ms, *τ*_3_ needs to be longer than 8ms to obtain stability (assuming *α* = 1).

Our main assumption is that the oscillation associated to the instability is to be avoided at all cost. While oscillations by themselves occur in many circumstances in neuroscience and have important functional roles, these particular oscillations here are uncontrollable and can not be stopped. Furthermore, the external input to the neuron has virtually no control over the oscillation’s phase, frequency or amplitude. Turning stimulation on or off hardly affects the neuron’s oscillatory activity, Fig 1B, bottom right. The oscillating state is almost the opposite of homeostasis, as it would be challenging for neurons to code information when oscillating like this. Especially if excitability is regulated through the insertion and removal of ion-channels, the oscillating state is also metabolically costly.

To warrant the absence of damped oscillations a similar criterion can be derived (Methods, Eq (13)) and in this example case *τ*_3_ needs to be longer than 220ms to avoid damped oscillations. In summary, for single neurons simple homeostatic control is stable even when it is very fast. Therefore, the stability of the homeostatic controller would not appear an issue for homeostasis of intrinsic excitability. Such very fast homeostasis might not even be desirable, because it will filter out components of the input slower than the homeostatic control. For instance in Fig 1B (top left), the neural response equals the stimulus with changes slower than ∼ 100ms filtered out.

### Global stability in non-linear neurons

While the above linear treatment covers the stability to small perturbations, stability to arbitrary perturbations is what ultimately matters. To analyze this the non-linearity of the f/I curve has to be taken into account. Unfortunately, non-linear stability analysis is generally much harder. Furthermore, stability proofs are typically sufficiency proofs, necessity proofs are rare. For the particular system under study, Eqs 1–3, it can be shown that it is *guaranteed* to be stable only when for all *x* (see Supplementary Information)

$$\begin{array}{c} 0<\frac{\tilde{g}\left(x\right)}{x}<\frac{\tau_{3}}{\tau_{3}^{0}} \end{array}$$ (4)

where $\tilde{g}(x)=g(x+x^{⋆})-g(x^{⋆})=g(x+x^{⋆})-r_{goal}$ is the f-I curve re-centered around the set-point ($\tilde{g}(0)=0$). The criterion thus becomes

$$\begin{array}{c} \tau_{3}>[\max\frac{\tilde{g}\left(x\right)}{x}]\frac{\tau_{1}\tau_{2}}{\tau_{1}+\tau_{2}} \end{array}$$

It is known as the Aizerman conjecture [27], and although not generally true and counter-examples do exist, it is known to hold for this particular 3 dimensional system [28]. The criterion replaces the local slope *α* with the slope of the line that goes through (*x* ^⋆^, *r*_*goal*_) and envelopes the f-I curve. Note that for a linear f-I curve this criterion equals the linear criterion, otherwise it is always more stringent than the linear criterion. We will further explore this criterion below.

### Local stability in recurrent networks

**Fig 2:**
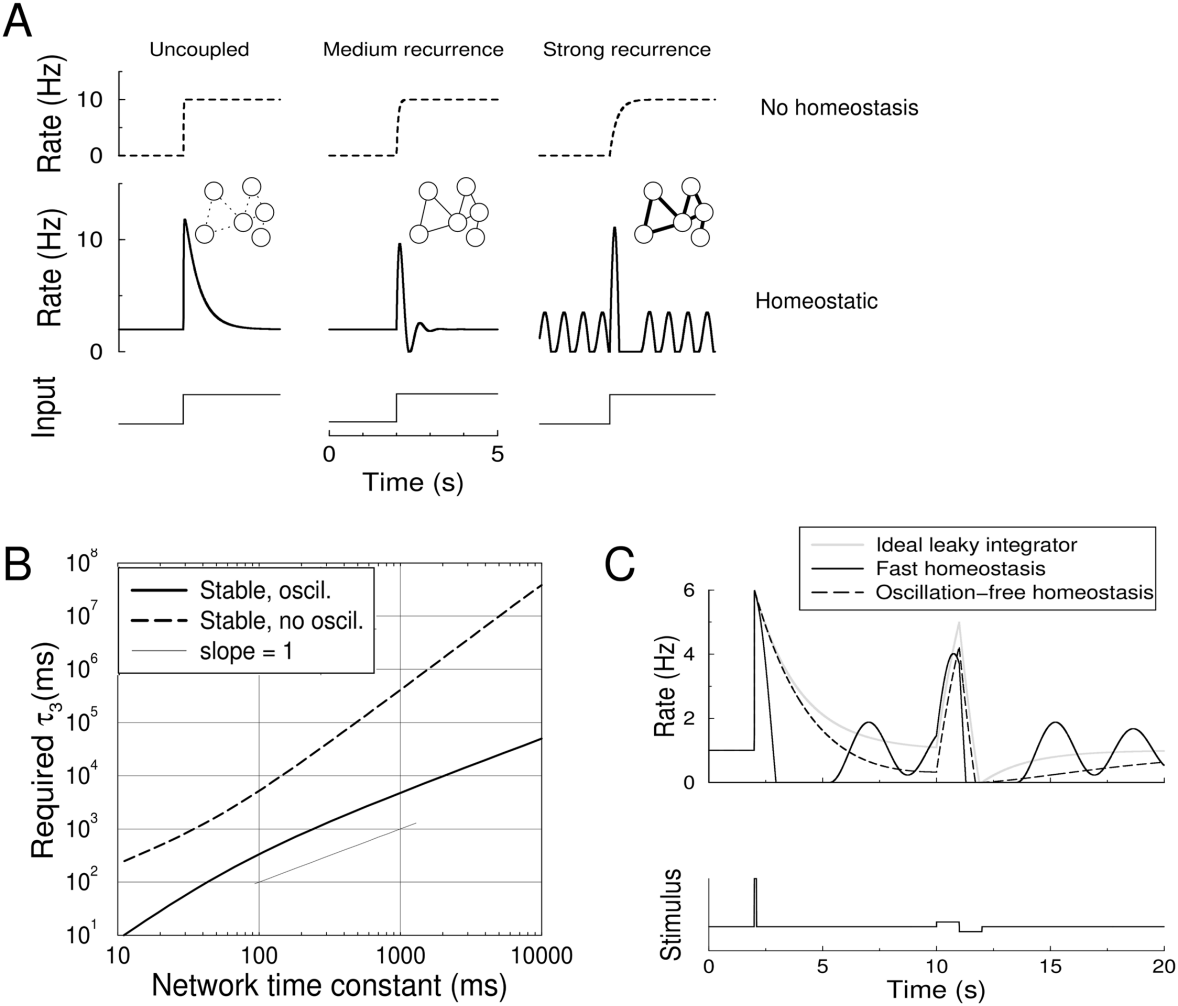
Homeostasis can destabilize activity in otherwise stable networks. A) Activity in a homeostatic network with varying levels of recurrence, but identical homeostatic parameters (*τ*_1_ = 10ms, *τ*_2_ = 50ms, *τ*_3_ = 500ms). Without homeostasis even the strongly recurrent network is stable (top row). With homeostasis, although the network in stable in the absence of synaptic coupling (left), with increasing recurrence the network shows increasing oscillatory activity (middle, *w*_*m*_ = 0.8), and becomes unstable for strong recurrence, leading to unabating oscillations (right, *w*_*m*_ = 0.95). B) The requirements on the homeostatic time-constant as a function of the recurrence of the network, expressed in terms of the network time-constant, which equals *τ*_1_/(1 − *w*_*m*_). Shown are the minimal value of *τ*_3_ to ensure stability, potentially with damped oscillations (solid curve) and the minimal value of *τ*_3_ for a stable firing rate without oscillation (dashed curve). C) The interference of homeostatic control with a neural integrator. The response of an ideal leaky-integrator with 1s time-constant (gray curve) to a pulse at 2s, and a bi-phasic pulse at 10 s. The response of a stable, but oscillatory homeostatic network is very different from the non-homeostatic case (black curve, *τ*_3_ = 7s). Only when the homeostasis is slow enough to be oscillation-free, the response approximates that of the ideal integrator (dashed curve, *τ*_3_ = 420s).

Next, we analyze the stability of homeostatic control in a *network* of neurons. For networks the conditions on homeostatic control are more stringent than for single neurons. In Fig 2A the population firing rate of a simulated network is plotted as the strength of the recurrent connections is increased while all other network and homeostasis parameters are fixed (left to right plot). Increasing the recurrent connections in the network leads to strong, persistent oscillations, while, importantly, without homeostasis the network is stable (top panels, dotted curves). To prevent instability the feedback needs to be slower in networks than for single neurons.

To analyze this, again first in the limit of small perturbations, we consider a network of *N* neurons connected with fast synapses via an *N* × *N* weight matrix *V*. In the absence of homeostasis the firing rate dynamics obeys

$$\begin{array}{c} \tau_{1}\frac{d}{dt}r_{1}\left(t\right)=-r_{1}\left(t\right)+G\left(Vr_{1}\left(t\right)+u\left(t\right)\right) \end{array}$$ (5)

where **r**_1_(*t*) is a *N*-dimensional vector containing all firing rates in the network, and **u**(*t*) is a vector of external input to the neurons in the network and *G* denotes the relation *g*() working on each of its elements. The recurrent feedback is contained in the term *V* **r**_1_(*t*). Linearized this becomes

$$\begin{array}{c} \tau_{1}\frac{d}{dt}r_{1}\left(t\right)=\left(W-I\right)r_{1}\left(t\right)+\alpha u\left(t\right) \end{array}$$

where we define the gain-scaled weight matrix *W* = *αV*, where *α* is again the slope of the f/I curve at the homeostatic set-point. After the linearization, the dynamics of these networks can be analyzed in terms of the eigen-modes. We denote the eigenvalues of *W* with *w*_*n*_. There are no a priori restrictions on *W*. The synapses can be excitatory or inhibitory, and one can impose Dale’s principle (which will affect the eigenvalue spectrum of the weight matrix [29], but does not change our results otherwise). For clarity we focus in the main text on cases where the eigenvalues are real, in the Methods the generalization to complex eigenvalues is presented. A typical example of a network where the eigenvalues are guaranteed to be real are symmetric networks that are used in many applications, such as noise filtering and evidence accumulation [30].

For such networks we define the largest eigenvalue, *w*_*m*_ = max(*w*_*n*_), as the *recurrence* of the network. The recurrent excitatory connectivity slows down the effective time-constant of a given mode [30, 31]. This can be seen by writing the equation for each mode as $\frac{\tau_{1}}{1-w_{n}}\frac{dr_{n}(t)}{dt}=-r_{n}(t)+\frac{\alpha }{1-w_{n}}u_{n}(t)$, from which the time-constant of a given mode is then identified as *τ*_1_/(1 − *w*_*n*_). The network time-constant is defined as the time-constant of the slowest mode, i.e. *τ*_1_/(1 − *w*_*m*_). Without homeostasis the network activity is stable as long as *w*_*m*_ < 1.

In the presence of homeostatic regulation, the system becomes 3*N*-dimensional. It is described by the rate of each neuron **r**_1_, its filtered version **r**_2_, and its threshold **r**_3_. The corresponding linearized differential equation is

$$\begin{array}{c} \frac{d}{dt}(\begin{array}{c} r_{1}\\ r_{2}\\ r_{3} \end{array})=M(\begin{array}{c} r_{1}\\ r_{2}\\ r_{3} \end{array})+(\begin{array}{c} \begin{array}{c} \frac{\alpha }{\tau_{1}}u\left(t\right)\\ 0 \end{array}\\ -\frac{1}{\tau_{3}}r_{goal} \end{array}) \end{array}$$

where *M* is now a block-matrix, given by

$$\begin{array}{c} M=(\begin{array}{ccc} \frac{1}{\tau_{1}}\left(W-I\right) & 0 & -\frac{\alpha }{\tau_{1}}I\\ \frac{1}{\tau_{2}}I & -\frac{1}{\tau_{2}}I & 0\\ 0 & \frac{1}{\tau_{3}}I & 0 \end{array}) \end{array}$$ (6)

We proceed as above to determine the stability of this system. In analogy with the single neuron case, there are three eigenvalues for the full system per eigenvector of *W*, so that we obtain 3*N* eigenvalues. In principle, one should now research the stability of each eigenvector of *W*. Yet the analysis can be simplified for networks with strictly real eigenvalues. In a network without homeostasis the most critical mode is the one with the largest eigenvalue. This also holds in networks with homeostasis: the network is stable if and only if this mode is stable (see Methods for proof). Thus, rather than analyzing the full network, we only need to analyze the stability of this most critical mode, which is given by a three dimensional system similar to the single neuron system studied above with the pre-factor of *r*_1_(*t*) on the right hand side as only modification,

$$\begin{array}{c} \tau_{1}\frac{dr_{1}\left(t\right)}{dt}=-\left[1-w_{m}\right]r_{1}\left(t\right)+\alpha \left[u\left(t\right)-\theta \left(t\right)\right] \end{array}$$ (7)

The other equations for homeostatic control, Eqs 2 and 3, remain unchanged. The resulting three dimensional system describes the dynamics of the critical eigenmode and its homeostatic variables. The stability is now determined by the roots of the polynomial

$$\begin{array}{c} \left(1-w_{m}+\tau_{1}\lambda \right)\left(1+\tau_{2}\lambda \right)\tau_{3}\lambda +\alpha =0 \end{array}$$ (8)

The network is again stable if all roots of this polynomial have a negative real part. Application of the Routh–Hurwitz criterion (Methods) yields the stability condition $\tau_{3}>\tau_{3}^{crit}$, where

$$\begin{array}{c} \tau_{3}^{crit}=\frac{\alpha }{1-w_{m}}[\frac{\tau_{1}\tau_{2}}{\tau_{1}+\left(1-w_{m}\right)\tau_{2}}] \end{array}$$ (9)

In Fig 2B we vary the integration time of the network by changing *w*_*m*_ and plot the values for *τ*_3_ required for stability. The minimal, critical value of *τ*_3_ is shown with the solid black curve. Eq (9) yields for (1 − *w*_*m*_)*τ*_2_ ≫ *τ*_1_ that *τ*_3_ ≳ *τ*_1_/(1 − *w*_*m*_)^2^, while for (1 − *w*_*m*_)*τ*_2_ ≪ *τ*_1_ this can be approximated as *τ*_3_ ≳ *τ*_2_/(1 − *w*_*m*_). When, for example, the network has an integration time-constant of 1s, *τ*_3_ needs to be at least 4.8s to prevent instability. If the network integration time-constant is 10s, this increases to 50s.

The linear stability analysis of networks with complex eigenvalues of the weight matrix is analogous, except that rather than only the largest, each eigenvalue needs to be checked (see Methods).

The stability to arbitrary perturbations again requires taking the non-linearity of the f/I curve into account. Nonlinear stability analysis of the network (Eq (5) supplemented with homeostasis) is presented in the Supplementary Information based on Lyapunov theory. The *τ*_3_, unfortunately a rather complicated expression, required for stability depends on the maximum slope of the f/I curve. The criterion value for *τ*_3_ is always slower than the value found using the linear theory, Eq (9), because, first, global stability implies local stability, but not vice versa, and secondly, the non-linear theory only provides a sufficiency condition for stability. We apply the criterion below.

### Oscillation-free homeostasis

The sustained oscillations associated to the instability are detrimental for neural information processing as they are uncontrollable and hinder information coding, yet are energetically expensive. Damped oscillations are less harmful. However, in particular for strongly recurrent networks, damped oscillations can interfere with the desired network response. As an illustration of this we show the response of an ideal leaky integrator, such as might be used for evidence integration in Fig 2C (gray curve). When rapid homeostasis is active, the response shows strong oscillations that occludes the network’s integrative properties (black curve). Only when homeostasis is made so slow that no damped oscillations occur (dashed curve), the response approximates that of the ideal integrator.

In recurrent networks the value of *τ*_3_ required to ensure homeostasis without damped oscillations is much larger than the value required to prevent persistent oscillation, compare dashed curve to solid curve in Fig 2B. Interestingly, as is shown in the Methods, for long integration times it increases as the square of the integration time (slope of 2 on the log-log plot). For example if the network integration time-constant is 1s, the minimal homeostatic time-constant is 420s to prevent transient oscillations. And if the network integration time-constant is 10s, a realistic value in for instance working memory networks [32], this values increases to 11hrs. In summary, in particular if an oscillation-free response is required, strongly recurrent networks with long time-constants require homeostasis many orders of magnitudes slower than single neurons and there is a strong dependence on the network time-constant.

### Variability and heterogeneity

To examine the generality of the results we included variability and heterogeneity in the model. First, we wondered whether heterogeneity in the time-constants, likely to occur in real neurons, could prevent the synchronous oscillations associated to the instability. Hereto we drew for each neuron the homeostatic time-constants from a gamma-distribution with an adjustable coefficient of variation (CV) and a given mean. To quantify the destabilizing effect of homeostasis, we defined the dimensionless *critical recurrence strength* *w*_*c*_. It is the maximal recurrence for which the network is still stable, possibly with damped oscillations. That is, *w*_*c*_ is the value at which the real part of the largest eigenvalue crosses zero. For networks without homeostasis, the critical recurrence is one, but homeostasis limits this to lower values.

**Fig 3:**
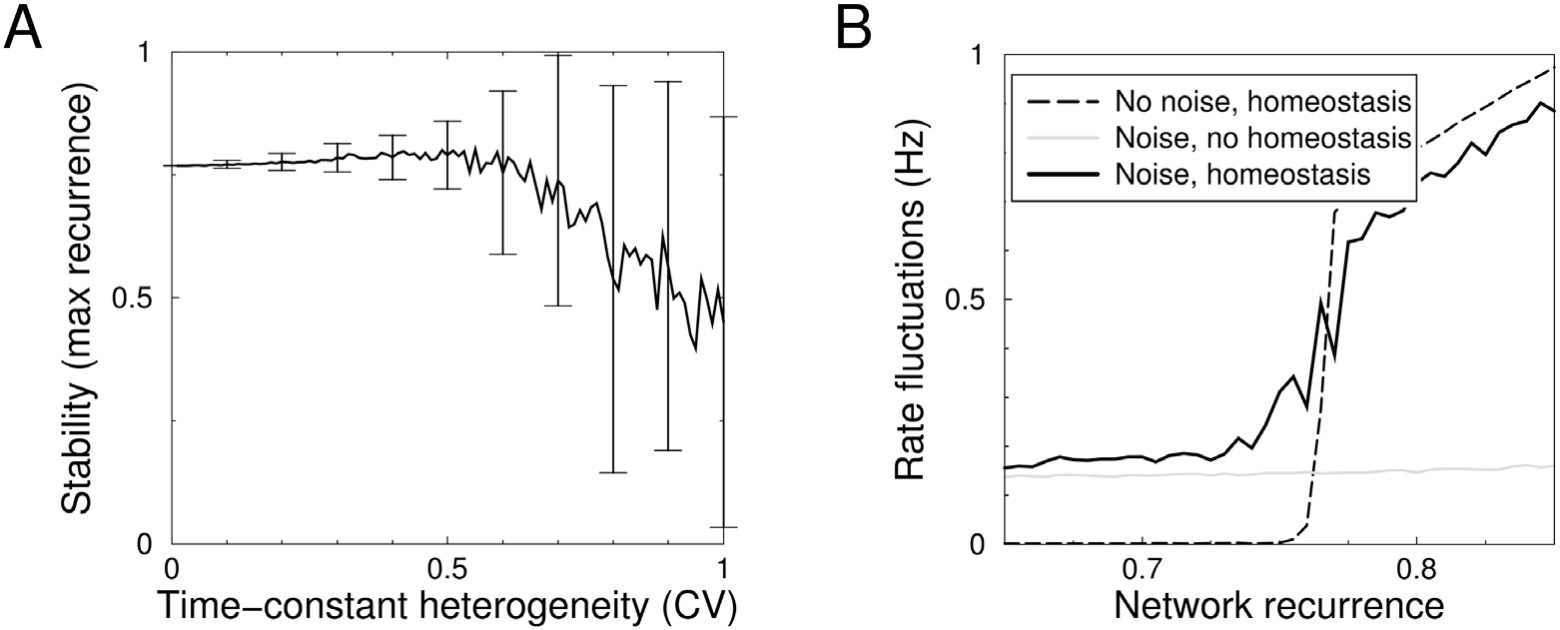
Effects of heterogeneity and noise on homeostatic stability of a network. A) The stability as a function of heterogeneity in the neurons’ homeostatic time-constants. The time-constants *τ*_1_, *τ*_2_, *τ*_3_ for each neuron were drawn from gamma-distributions with means 10, 50 and 100ms, respectively, and a CV given by the x-axis. The curve represents the mean maximal recurrence allowed to ensure a stable system. It decreases with heterogeneity. Error bars represent the standard deviation over 1000 trials. Simulation of 10 neurons, connected with a random, fixed weight matrix. B) Noise does not ameliorate instability. The fluctuations in the population firing rate, due to both noise and oscillations, are plotted as a function of the network recurrence. Without noise, fluctuations are only present when the recurrence exceeds the critical value (dashed curve). With noise, the fluctuations are already present in the stable regime and increase close to the transition point (solid curve). The homeostasis in the system amplifies noise compared to the non-homeostatic system (grey line). Homeostatic time-constants were 10, 50 and 100ms.

Stability is again determined by the stability matrix of Eq (6), however, in the heterogeneous case the dimension reduction is not possible and the spectrum of the full matrix was examined. When the CV is zero, all neurons have the same set of time-constants and the stability corresponded to that of the homogeneous networks. As the heterogeneity increased, the average maximal allowed recurrence first increased slightly after which it decreased, Fig 3A. Moreover, as can be seen from the error bars, for a given realization of the time-constants, the stability can either be higher or lower than that of the homogeneous network. Hence random heterogeneity of the time-constants does not robustly lead to increased stability. The effect of heterogeneity on the transition between the damped oscillatory and oscillation-free regime is similar.

Next, we added noise to the neurons and analyzed how this affected the transition to instability. The noise might potentially have a stabilizing effect by de-synchronizing the population. Gaussian noise with a correlation time of 1ms and a standard deviation equivalent to 0.1Hz was added to the input. We measured the fluctuations as the standard deviation of the population firing rate once the system had reached steady state, Fig 3B. These fluctuations comprise both the effect of noise and the periodic oscillations caused by the instability. Without noise, fluctuations are absent when the recurrence is less than the critical amount (Fig 2A), and are strong above this point, Fig 3B (dashed curve). With noise, fluctuations are always present (solid curve) and increase close to the transition to instability. Above the transition point the fluctuations are similar to the noise-free model. In a network with homeostasis the resulting fluctuations were always larger than without (gray line). The reason is that in the homeostatic network the noise is continuously exciting a damped resonant system, amplifying the fluctuations. Importantly, the amount of recurrence at which the transition to the unstable regime occurs, does not shift with noise, implying that noise does not increase stability. Rather the opposite happens. Already in the approach to instability (around a recurrence of 0.75), homeostasis increases the fluctuations in the population firing rate (black curve diverges from gray curve).

### Spiking networks

Next, we compared the theory to simulations of networks of spiking neurons (see Methods). The connection strength was such that the network was stable and noise was injected to all neurons to prevent population synchrony. The homeostatic control was implemented exactly as above: the average rate *r*_2_(*t*) was extracted by filtering the spikes (*τ*_2_ = 50ms), and this was fed into the integrator as above. The homeostatic target rate was set to 4Hz.

**Fig 4:**
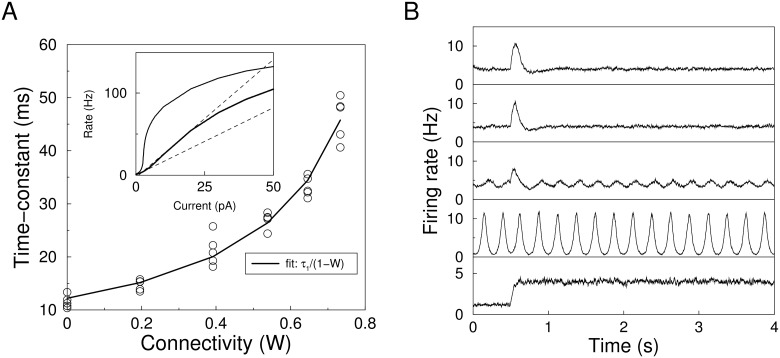
Homeostatic regulation in a network of integrate-and-fire neurons. A) The effective time-constant of the network as a function of recurrent connection strength. Circles denote simulation results and the curve is the fitted relation *τ*_1_/(1 − *w*_*m*_). Inset shows the f-I curve of the unconnected network (lower curve), the slope at the set-point (lower line) and slope of the envelop (upper line). F-I curve of the connected network (upper curve) is shown for comparison. B) Example population response to step stimuli for varying values of *τ*_3_ corresponding to, from top-to bottom: *τ*_3_ = 380ms (stable according to Aizerman criterion); *τ*_3_ = 240ms (empirically stable); *τ*_3_ = 200ms (edge of instability); *τ*_3_ = 64ms (linear criterion). Bottom panel: network without homeostasis.

In this asynchronous regime, the population firing rate of the spiking network can be reasonably approximated by the rate equation with a non-linear f-I curve (Eq (1)) and recurrent feedback. In order to be able to compare the spiking network to the theory we turned homeostasis off and gave small step stimuli to the network and measured how quickly the firing rate equilibrated as a function of the connection strength, Fig 4A. In the rate model this equilibration time is *τ*_1_/(1 − *w*_*m*_). A fit to this relation gave *τ*_1_ ≈ (11.5±1.5)ms and also yielded the proportionality between the synaptic strength and *w*_*m*_, which we calibrated as above so that *w*_*m*_ = 1 corresponds to the critical amount of recurrence in the linearized model without homeostasis. As networks close to critical recurrence are slow and difficult to simulate, we used a value of *w*_*m*_ = 0.6, so the required homeostatic time-constants are fairly short.

The linear stability criterion, Eq (9) yields that when *τ*_3_ ≥ 64ms the network should be stable. However, the simulated network is less stable than the linear criterion predicts. The network shows strong oscillations for such rapid homeostasis, Fig 4B, second plot from below. In simulations a minimal value of *τ*_3_ around 240ms was needed to stabilize the network.

To include the effect of the non-linearity we first used the Lyapunov-based criterion (see above and S1 Text) which yielded *τ*_3_ ≥ 1200ms. To see if a tighter bound was possible, we applied the Aizerman criterion to the slowest mode. Note that this is strictly only valid for a 3 dimensional system and not the 3*N* dimensional system. Thus we assume that the eigenmodes of the system do not or only weakly couple. Under this assumption stability is guaranteed when

$$\begin{array}{c} \tau_{3}^{aiz}=\frac{\alpha \beta }{1-\beta w_{m}}[\frac{\tau_{1}\tau_{2}}{\tau_{1}+\left(1-\beta w_{m}\right)\tau_{2}}] \end{array}$$ (10)

where $\beta ={\text{max}}_{x}\left(\frac{\tilde{g}(x)}{x}\right)/\alpha$. In other words in the Aizerman criterion, the slope at the origin of $\tilde{g}(x)$ is replaced by the slope of the linear envelope. The parameters *α* and *β* are extracted from the f-I curve of the unconnected network, Fig 4A, inset. When applied to our simulations, the criterion leads to a value of $\tau_{3}^{aiz}=380$ms, which is not far from the minimal value found numerically. This indeed leads to stable homeostasis, Fig 4C, top.

### Cascaded homeostatic control

The above results assumed a simple controller with only three components in the feedback loop, *r*_1_, *r*_2_, and *r*_3_, but homeostatic control of excitability has many intermediate stages, for instance synthesis, transport and insertion of ion-channels are likely involved. Therefore we asked how the stability of homeostatic control changes with longer feedback cascades. Our intuition was that adding more elements to the feedback cascade would slow down the feedback, and therefore would increase stability. However, we found that adding more filters actually de-stabilizes the network.

**Fig 5:**
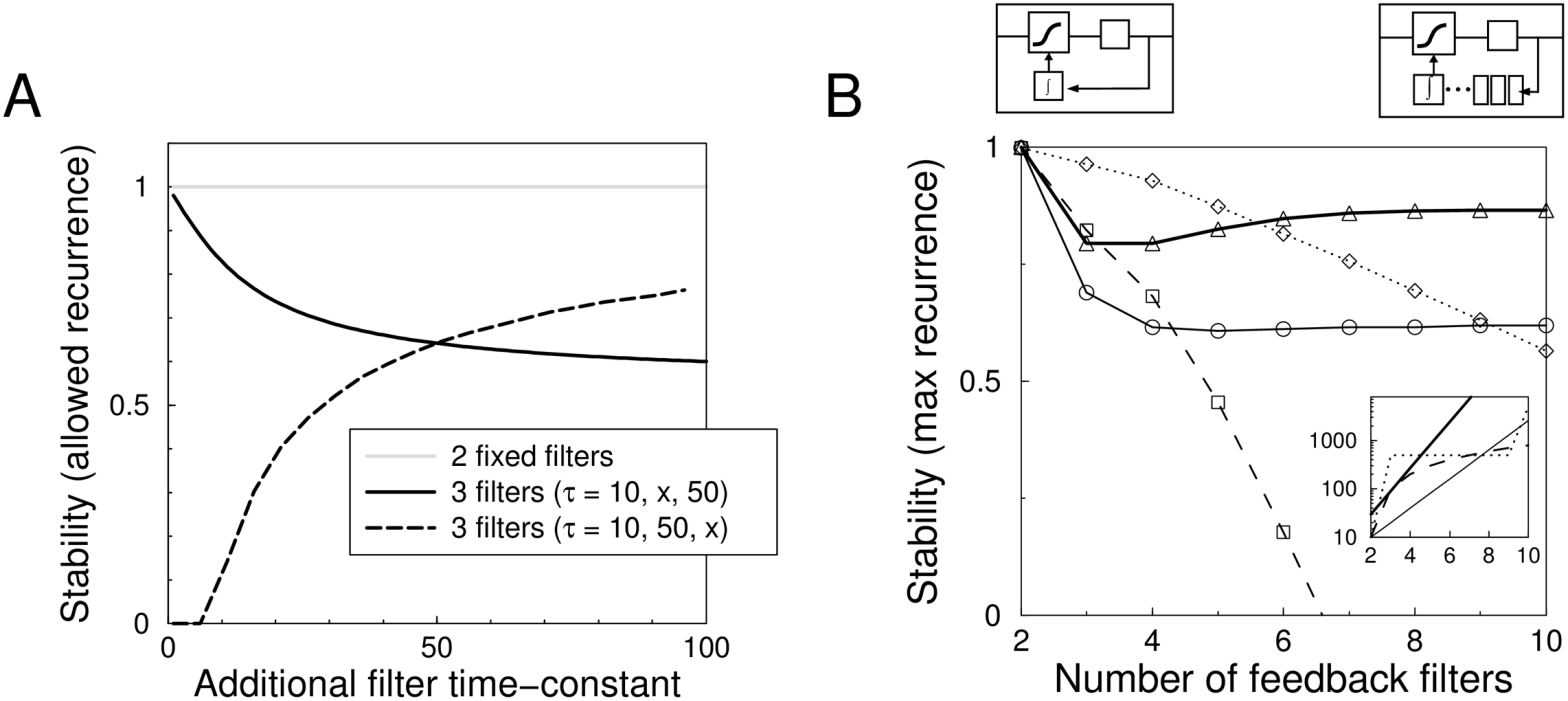
Networks with longer feedback cascade are less stable. A) The effect of adding a third filter to a two filter cascade. The stability is expressed as the maximum recurrence allowed in the network before it becomes unstable (transient oscillations allowed). The system with three filters is always less stable than the two filter system. The time-constants were set *τ*_1_ = 10, *τ*_2_ = 50 in the case of two filters, and *τ*_1_ = 10, *τ*_2_ = 50, *τ*_3_ = *x*, as well as *τ*_1_ = 10, *τ*_2_ = *x*, *τ*_3_ = 50 for the three filter case. B) Stability versus the number of filters for various filter cascades. As a function of filter number, time-constants were set linear 10, 20, 30, 40, … (dashed), constant with slow final integrator 10, 500, 500, …, 500, 5000 (dot-dashed), or exponential 10, 20, 40, … (solid) and 10, 30, 90, …ms (thick solid). The inset show the time-constants for a cascade with 10 filters for the various cases. Except for the last case, stability decreases with the number of filters.

We first simplify our model from three to two filters, and analyze what happens to the critical amount of network recurrence if we add a third filter, Fig 5A. With two filters (*τ*_1_ = 10*ms*, *τ*_2_ = 50*ms*) the critical recurrence is one, the same as for a network without homeostasis (gray curve). The addition of a third filter, such that the time-constants are (*τ*_1_, *τ*_2_, *τ*_3_) = (10, 50, *τ*) is destabilizing even if the third filter has a time-constant slower than any other time-constant (dashed curve). Only for a very long time-constant it had no detrimental effect. Alternatively, one can add an intermediate filter, such that the time-constants are (*τ*_1_, *τ*_2_, *τ*_3_) = (10, *τ*, 50). Also this is destabilizing (solid line). In this case the destabilizing effect can be minimized by taking *τ* as short as possible. The filter then has a negligible effect, and the system resembles the two filter system again.

More generally, assuming that there is no intermediate feedback between the filters and that each element can be approximated by a linear filter, our formalism can be extended to an arbitrary number of intermediate elements in the feedback loop. Suppose that we have *K* filters, each with its own time-constant *τ*_*k*_. The threshold is taken from the *K*-th filter, i.e. *θ*(*t*) = *r*_*K*_(*t*). We thus have for the linearized system

$$\begin{array}{ccc} \tau_{1}\frac{dr_{1}\left(t\right)}{dt} & = & -\left[1-w_{m}\right]r_{1}\left(t\right)+u\left(t\right)-r_{K}\left(t\right)\\ \tau_{k}\frac{dr_{k}\left(t\right)}{dt} & = & -r_{k}\left(t\right)+r_{k-1}\left(t\right)\;k=2\ldots K-1\\ \tau_{K}\frac{dr_{K}\left(t\right)}{dt} & = & -r_{goal}+r_{K-1}\left(t\right) \end{array}$$

The corresponding characteristic polynomial in this case is

$$\begin{array}{c} 1+\lambda \tau_{K}\left(1-w_{m}+\lambda \tau_{1}\right)\;\prod_{k=2}^{K-1}(1+\lambda \tau_{k})=0 \end{array}$$ (11)

This expression is invariant to permutations of the time-constants *τ*_2_, …, *τ*_*K*−1_. The stability is again determined by the real part of the solutions to the polynomial. As analytic results such as Routh-Hurwitz analysis, quickly grow in complexity for an increasing number of filters, we solve the polynomial numerically.

As the time-constants or even the number of steps in the homeostatic feedback in neurons is not known, we examined the stability with various hypothetical settings of the additional filters, Fig 5B. When the time-constants were set linearly increasing as *τ*_*i*_ = 10, 100, 200, 300, …ms, the stability decreased most strongly as the number of stages *K* increased (dashed curve). Using *τ*_*i*_ = 10, 500, 500, …, 500, 5000, stability decreased also with the number of filters (dot-dashed curve). When the time-constants were set exponentially as *τ*_*i*_ = 10, 20, 40, 80 … stability decreased when using only few filters, and leveled off with more filters (black curve). With a stronger exponential increase *τ*_*i*_ = 10, 30, 90, 270 … the stability reached a minimum for 4 filters and then slightly increased to a constant level (thick black curve). Thus in general addition of filters does not lead to stabilization of the system. This result is not dependent on these particular time-constants, also when for instance *τ*_3,4,…*K*_ are orders of magnitude slower than *τ*_1_ and *τ*_2_, the destabilization occurs.

We wondered what choice of time-constants will be most stable for a given number of filters. Suppose a cascade where the time-constant of the firing rate *τ*_1_ and of the threshold setting *τ*_*K*_ are fixed. In analogy with the three filter network, setting the time-constants of the intermediate stages as short as possible is the most stable configuration. Even adding an intermediate filter with a time-constant much slower than *τ*_*K*_ will not stabilize the system. The intuition behind these results is that not only the speed of the feedback matters, but its phase delay matters as well. With sufficient filtering the negative homeostatic feedback will be out of phase with the firing rate, amplifying perturbations. This effect is similar to the typically destabilizing effect of delays in control theory.

Next we use Eq (11) to study how network recurrence and cascade depth interact. As an example, consider the case where *τ*_1_ = 10ms, *τ*_2_ = 20ms, and *w*_*m*_ = 0.99. If *w*_*m*_ increases to 0.995 the required *τ*_3_ doubles from 4.7 to 9.7 s. Alternatively, adding an intermediate filter with a time-constant of 50ms also approximately doubles the required time-constant of the integrator to 9.5s. When we increase both *w*_*m*_ and increase the number of filters, the required *τ*_3_ quadruples to 19.5s. Thus the effect of recurrence and cascade length are complementary.

### Parallel controllers

**Fig 6:**
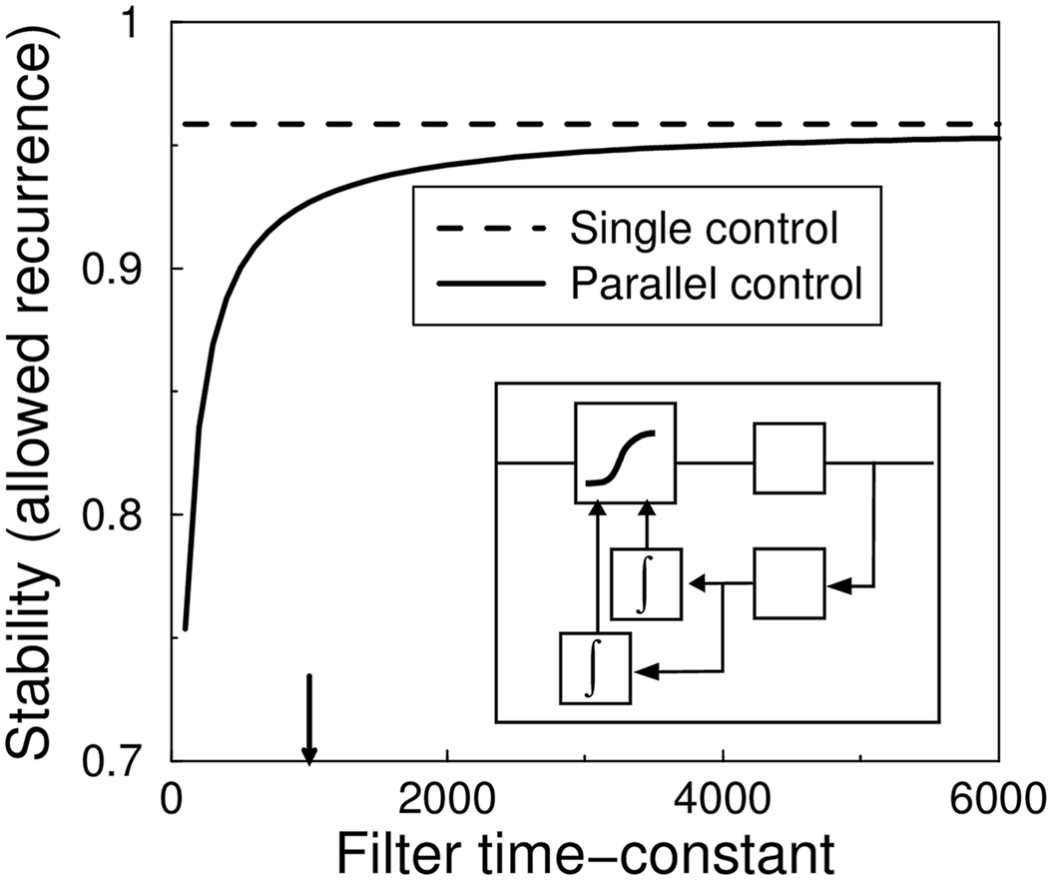
Parallel controllers do not lead to increased stability. The maximal recurrence is plotted against the time-constant of the second feedback loop. The *τ*_3_ time-constant of the system with single feedback is indicated by the arrow. The system with the extra feedback loop (solid curve) is always less stable than the system with a single feedback loop (dashed line), even if the additional feedback is much slower than the original one. The control loop is shown in the inset.

One can wonder if stability can be rescued in another way. For instance, it is not unreasonable to assume that biology uses multiple, parallel homeostatic regulators. While a general theory of such systems is lacking, some cases can be incorporated in our framework, for instance if multiple feed-backs use the same error signal, stability is determined by the quickest feedback. An addition of a parallel feedback, even if it is slower can only destabilize the system. The stability can be analyzed using the above techniques, adding the extra controller to the feedback-loop. As a technicality, because the system is invariant to the division of labor between the two feedback loops, the stability matrix gains a zero eigenvalue, which can be safely ignored. The system with parallel controllers is always less stable than the system with a single controller, even if the second controller is slower than the first one, Fig 6.

## Discussion

We have systematically analyzed instabilities in the neural activity that arise from homeostasis of intrinsic excitability. In the worst case, homeostasis can lead to continuous oscillations of the activity. Homeostasis can also give rise to damped oscillations, which are probably less disastrous to information processing, provided the oscillations do not persist too long. To our knowledge such damped oscillations in the homeostatic response have not been observed experimentally, although averaging of experimental data could have obscured their detection. Nevertheless, we think that they are unlikely to occur in biology because substantial cost is involved in alternating up-down regulation of excitability, and because the homeostatic control can strongly interact with the network activity (Fig 2C).

Our control theoretic framework for homeostasis sets constraints on homeostatic control to prevent either form of instability and we have focused on three contributions to the stability: recurrent network interactions, depth of the feedback loop, and non-linearities. First, we find that a typical single neuron model with just a few filters in the feedback loop has no stability issues even when the homeostatic control is very fast. However, this is no longer true when network interactions are included. The stronger the recurrence of the network, the slower the feedback needs to be. Networks with time-constants on the order of seconds have been proposed to explain sensory evidence integration, decision making and motor control [32–34]. For homeostasis to be oscillation-free, the minimal homeostatic time-constant scales quadratically with the network time-constant. Thus in particular for networks with slow dynamics, the required homeostasis can become of the order of hours, a value comparable to experimentally observed homeostatic action [13, 15, 20, 21].

Stability typically decreases further when the number of stages in the feedback loop increases, Fig 5. This effect complements the effect of the recurrence, so that for recurrent networks consisting of neurons with long homeostatic cascades, even slower homeostasis is required. The instability can not be prevented by including heterogeneity or adding noise to the system and is also found in spiking network simulations.

The above results are mainly based on the linearized system, which describes stability to small perturbations. In addition, we have derived the condition for stability to arbitrary size perturbations in the case that the f-I curve is non-linear. The non-linear f-I curve limits the minimal homeostatic time-constant even further. Ideally, one would like to know the stability requirements for any given non-linear homeostatic controller. However, only in a very limited number of cases extensions of mathematical results to either multiple non-linearities in the control loop or to higher dimensional systems (i.e. with longer feedback cascades) are known. These are topics of current control theory research.

Stability of homeostatic control has been the main consideration in this study. This is of course of utmost importance biologically, but it is unlikely to be the only criterion. There can also be cases where rapid acting homeostasis is needed. For instance, one might want to minimize periods of prolonged hyperactivity, while in a recent study fast *synaptic* homeostasis was required to counter synaptic plasticity [35]. It suggests that homeostatic control is constrained “from below and from above”, and therefore more finely tuned than previously thought.

Unfortunately data on the time-course of the homeostasis of intrinsic excitability, its mediators and regulation cascade is limited, hindering a direct comparison of data to our analysis. Nevertheless, a number of predictions follows from this work: we predict homeostasis to be slower in brain regions with strong recurrent connections and long network integration times. Secondly, we predict that intermediate steps in the homeostatic feedback cascade are rapid so as to prevent instability.

A recent complementary study examined homeostatic control for a network with separate excitatory and inhibitory populations and a shallow feedback loop (*K* = 2) and found as the only requirement for stability that the homeostasis of excitatory neurons is at least as fast as that of inhibitory neurons [22]. When excitation and inhibition are subject to equally fast homeostasis, the system is identical to the one studied here. As for these shallow feedback loops the homeostasis is always stable (our Fig 5A), no constraint on the speed of homeostasis relative to the neural and network timescales arises in that study. It should be possible to use our framework to extend those two population results to deeper feedback cascades. Other targets for extension and application of this study include excitatory/inhibitory balanced networks, controllers with parallel slow and fast components, as well as models that include dynamical synapses. Also the interaction with ‘Hebbian’ modification of the intrinsic excitability [36] will be of interest. Finally, these results might be important for other regulatory feedback systems such as synaptic homeostasis and spike frequency adaptation.

## Methods

### Stability in recurrent network

In the main text we state that stability of a homeostatic network is determined by the stability of the mode with the largest eigenvalue. Here we prove that if the reduced linear (3D) model based on the largest eigenvalue is stable, then so is the full (3N dimensional) linearized network model. Given the interaction matrix *M* of the full network, Eq (6), it is easy to show that the eigenvectors of the matrix *M* have the form $\left(\begin{array}{c} \begin{array}{c} {\text{e}}_{n}\\ \alpha_{n}{\text{e}}_{n}\\ \beta_{n}{\text{e}}_{n} \end{array} \end{array}\right)$, where **e**_*n*_ is an eigenvector of the *W* matrix, and *α*_*n*_ and *β*_*n*_ are complex numbers. This means that the filtered firing rates (the vectors **r**_2_ and **r**_3_) follow the firing rates **r**_1_ with a phase lag and arbitrary amplitude. We assume that *N* × *N* matrix *W* is symmetric so that it is diagonizable by an orthogonal matrix, that is *W* = *U* ^*T*^ *DU*, where *D* is a diagonal matrix with the eigenvalues *w*_*n*_ on the diagonal and *UU* ^*T*^ = *I*. We analyze *M* in the eigenspace of *W* using the matrix *U*_3*N*_ = *U* ⊗ *I*_3_, where ⊗ is the Kronecker product. In these coordinates $\bar{M}=U_{3N}MU_{3N}^{T}$ and equals

$$\begin{array}{ccc} \bar{M} & = & (\begin{array}{ccc} \frac{1}{\tau_{1}}\left(D-I\right) & 0 & -\frac{1}{\tau_{1}}I\\ \frac{1}{\tau_{2}}I & -\frac{1}{\tau_{2}}I & 0\\ 0 & \frac{1}{\tau_{3}}I & 0 \end{array}). \end{array}$$

In these coordinates, there is no interaction between the various eigenmodes. The stability of each mode is given by Eq (9). Because the factor (1 − *w*_*n*_) is positive and minimal for *w*_*n*_ = *w*_*m*_, stability of the eigenmode with eigenvalue *w*_*m*_ implies stability for all other modes for which *w*_*n*_ ≤ *w*_*m*_.

The stability condition is found from the Routh–Hurwitz stability criterion [25]. It states that the third order polynomial $\sum_{i=0}^{3}c_{i}\lambda^{i}=0$ has exclusively negative roots when 1) all the coefficients *c*_*i*_ are larger than zero, and 2) *c*_0_ *c*_3_ < *c*_1_ *c*_2_. Applied to homeostatic control this yields Eq (9).

### Non-symmetric networks

The analysis can be extended to networks with non-symmetric weight matrices. Symmetry of *W* implies that the eigenvalues of the matrix *W* are real. For non-symmetric *W*, the eigenvalues are no longer guaranteed to be real but can be complex. The Routh-Hurwitz criterion needs now to be applied after splitting the real and imaginary parts of the polynomial. The conditions that guarantee negative real parts for the solutions of the polynomial *λ* ^3^ + *c*_1_ *λ* ^2^ + *c*_2_ *λ* + *c*_3_ = 0 with complex coefficients *c*_*i*_ are [37]: 1) ℜ(*c*_1_) > 0, 2) $ℜ(c_{1})ℜ(c_{1}{\bar{c}}_{2}-c_{3})-ℑ{\left(c_{2}\right)}^{2}>0$, and 3) $[ℜ(c_{1})ℜ(c_{1}{\bar{c}}_{3})-ℜ{\left(c_{3}\right)}^{2}][ℜ(c_{1})ℜ(c_{1}{\bar{c}}_{2}-c_{3})-ℑ{\left(c_{2}\right)}^{2}]-{\left[ℜ(c_{1})ℑ({\bar{c}}_{1}c_{3})-ℜ(c_{3})ℑ(c_{2})\right]}^{2}>0$, where $\bar{c}$ denotes the complex conjugate of *c*, and ℜ and ℑ the real and imaginary parts. In this case one has *c*_1_ = 1/*τ*_2_ + (1 − *w*_*n*_)/*τ*_1_, *c*_2_ = (1 − *w*_*n*_)/*τ*_1_ *τ*_2_, *c*_3_ = 1/*τ*_1_ *τ*_2_ *τ*_3_, where *w*_*n*_ is the complex eigenvalue. Splitting the real and imaginary parts as *w*_*n*_ = *w*_*r*_ + *iw*_*i*_, these conditions combine to the condition $\tau_{3}\geq \tau_{3}^{cc}$ with

$$\begin{array}{c} \tau_{3}^{cc}=\frac{1}{1-w_{r}}\frac{\tau_{1}\tau_{2}\left[\tau_{1}+\left(1-w_{r}\right)\tau_{2}\right]+\frac{1}{2}\tau_{2}^{3}w_{i}^{2}\left[1+\sqrt{1+4\tau_{1}\left(1-w_{r}\right)/\left(\tau_{2}w_{i}^{2}\right)}\right]}{{\left[\tau_{1}+\left(1-w_{r}\right)\tau_{2}\right]}^{2}+w_{i}^{2}\tau_{2}^{2}} \end{array}$$ (12)

In contrast to the case of symmetric *W*, these conditions have to be checked for all *N* eigenvalues of *W*. By taking the limit of infinite *w*_*i*_ it can be shown that stability is guaranteed for any complex *w*_*n*_ when *τ*_3_ > *τ*_2_/(1 − *w*_*r*_), which is more stringent than the condition given in Eq (9). Under this condition any network, including non-symmetric ones, is guaranteed to be stable to small pertubations.

### Oscillation-free response

To guarantee an oscillation-free response of the network, the eigenvalues need to be negative and real. For a given *w*_*n*_ this implies that all the solutions of the polynomial

$$\begin{array}{c} P\left(\lambda \right)=\left(1-w_{n}+\tau_{1}\lambda \right)\left(1+\tau_{2}\lambda \right)\tau_{3}\lambda +1 \end{array}$$

have to be real. As in our analysis above, the largest eigenvalue of *W* is the most critical one so that we only need to study the case *w*_*n*_ = *w*_*m*_.

The polynomial is negative for large, negative *λ* and positive for large, positive *λ*. For all solutions to be real, the polynomial has to dip down after the first zero-crossing and cross zero again, after which it crosses the x-axis a final time. The condition on the minimum of the dip, given by *P*′(*λ*_*c*_) = 0 and *P*′′(*λ*_*c*_) > 0, is that it should be below zero, i.e. *P*(*λ*_*c*_) < 0. This yields the condition $\tau_{3}\geq \tau_{3}^{co}$ with

$$\begin{array}{c} \tau_{3}^{co}=\frac{1}{{\left(1-w_{m}\right)}^{2}{\left(\tau_{1}-\tau_{2}^{\prime }\right)}^{2}}[\left(\tau_{1}-2\tau_{2}^{\prime }\right)\left(2\tau_{1}-\tau_{2}^{\prime }\right)\left(\tau_{1}+\tau_{2}^{\prime }\right)+2{\left(\tau_{1}^{2}-\tau_{1}\tau_{2}+\tau_{2}^{2}\right)}^{3/2}] \end{array}$$ (13)

where we defined $\tau_{2}^{\prime }=(1-w_{m})\tau_{2}$. In the limit of strong recurrence $\tau_{3}^{co}=\frac{4}{\tau_{1}}{\left(\frac{\tau_{1}}{1-w_{m}}\right)}^{2}$, which implies that the required time-constant *τ*_3_ scales quadratically with the network time-constant, *τ*_1_/1 − *w*_*m*_.

### Spiking network simulations

A population of 16000 linear integrate-and-fire neurons was coupled with a 2% connection probability via excitatory synapses modeled as exponentially decaying conductances (5ms synaptic time-constant). It is possible to add inhibitory connections to the network, but as long as the network remains in the mean-driven regime this should not affect the results. The membrane voltage of each neuron obeyed $\tau_{mem}\frac{dV(t)}{dt}=-V(t)+V_{rest}+RI(t)$, where *t*_*mem*_ = 20ms, *V*_*rest*_ = −60mV and *R* = 1MΩ. In addition, upon reaching the threshold (*V*_*thr*_ = −50mV) the voltage reset (*V*_*reset*_ = *V*_*rest*_, 5ms refractory period). The current *I* consisted of recurrent input, external drive and homeostatic bias, *I*(*t*) = *g*_*e*_(*t*)(*V*(*t*) − *E*_*e*_)+*I*(*t*) − *hr*_3_(*t*). The factor *h* converts the filtered firing rate *r*_3_ to a current and sets the strength of the homeostatic control. It was set to 1 *pA*/*Hz*. The homeostatic control was implemented as in the rate based networks: the average rate *r*_2_(*t*) was extracted by filtering the spikes (*τ*_2_ = 50ms), and this was fed into the integrator. The homeostatic target rate was set to 4Hz. The external current *I*(*t*) contains both stimulation and a Gaussian white noise term (*σ* = 75pA) to prevent population synchrony.

## Supporting Information

S1 Text Derivation of homeostatic stability criteria for non-linear networks. The derivation of stability when the f/I curve is non-linear. Both for the full (3N-dimensional) network, as well as for the system reduced to the slowest eigenvalue.
(PDF)
