# Supplementary material for: Stability of Neuronal Networks with Homeostatic Regulation
Source: PLoS Comput Biol. 2015 Jul 8;11(7):e1004357. doi: 10.1371/journal.pcbi.1004357 (PMC4495932; doi:10.1371/journal.pcbi.1004357)
Supplement: S1 Text — The derivation of stability when the f/I curve is non-linear. Both for the full (3N-dimensional) network, as well as for the system reduced to the slowest eigenvalue. (PDF) [file pcbi.1004357.s001.pdf]

Supplementary Information: Stability of neuronal networks with homeostatic regulation

Daniel Harnack, Miha Pelko, Antoine Chaillet, Yacine Chitour, Mark C.W. van Rossum

May 24, 2015

Here we 1) derive stability conditions for the network with a non-linear f/I curve, and 2) the tighter stability criterion which be obtained by considering only the slowest mode of the system. Qualitatively, for the 3-dimensional system a criterion exists based on the envelope of the non-linearity. For the full network, the criterion is based on the maximum slope of the non-linearity and this is leads to a less tight bound.

1 $3N$ dimensional nonlinear model

The rate-based model of the full network under concern is

$$\dot{r}_1 = -\frac{r_1}{\tau_1} - \frac{1}{\tau_1} G(r_3 - W r_1 + u) \tag{1a}$$

$$\dot{r}_2 = -\frac{r_2}{\tau_2} + \frac{r_1}{\tau_2} \tag{1b}$$

$$\dot{r}_3 = \frac{r_2}{\tau_3} - \frac{r_{goal}}{\tau_3} \mathbb{1}_N, \tag{1c}$$

where $r_1, r_2, r_3, u \in \mathbb{R}^N$, $G : \mathbb{R}^N \to \mathbb{R}^N$ satisfies $G_i(x) = -g(-x_i)$ for some strictly increasing bounded function $g : \mathbb{R} \to \mathbb{R}$, $W \in \mathbb{R}^{N \times N}$ is a symmetric matrix of interconnection gains, and $\mathbb{1}_N$ denotes the vector of $\mathbb{R}^N$ with all entries equal to 1.

For a constant input $u = u^* \in \mathbb{R}^N$ and a given $r_{goal} \in \mathbb{R}$ satisfying $g(-\infty) < r_{goal} < g(+\infty)$, the equilibrium r^* of (1) is unique and is given by

$$r_1^* = r_{goal} \mathbb{1}_N \tag{2a}$$

$$r_2^* = r_{goal} \mathbb{1}_N \tag{2b}$$

$$r_3^* = -u^* + r_{goal} W \mathbb{1}_N + g^{-1}(-r_{goal}) \mathbb{1}_N, \tag{2c}$$

Let $x := r - r^* \in \mathbb{R}^{3N}$, then the system can be written as

$$\dot{x}_1 = -\frac{x_1}{\tau_1} - \frac{1}{\tau_1} \tilde{G}(x_3 - W x_1) \tag{3a}$$

$$\dot{x}_2 = -\frac{x_2}{\tau_2} + \frac{x_1}{\tau_2} \tag{3b}$$

$$\dot{x}_3 = \frac{x_2}{\tau_3}, \tag{3c}$$

where $\tilde{G} : \mathbb{R}^N \to \mathbb{R}^N$ is defined as

$$\tilde{G}(z) := G(z - W r_1^* + r_3^*) + r_1^*, \quad \forall z \in \mathbb{R}^N.$$

Notice that $\tilde{G}(0_N) = 0_N$ and $D\tilde{G}(0) = h I_N$ with

$$h := g'\left(g^{-1}(-r_{goal})\right), \tag{4}$$

so that the system (3) has now its equilibrium conveniently at zero.

1.1 Sufficient conditions for global stability of the nonlinear network

We will we assume that g has a bounded derivative. We thus set $h_m := \sup_{x\in\mathbb{R}} g'(x)$. set $G_m := \sup_{x\in\mathbb{R}^N} \|\tilde{G}\|$ and $\bar{\omega}_m$ as the maximum over the absolute values of the eigenvalues of W.

In order to derive a sufficient condition for the stability of System (3), one rewrites the dynamics of x_1 in Eq. (3a) as

$$\tau_1\dot{x}_1 = -x_1 - \tilde{G}(x_3) + \Delta G(x_3, x_3 - Wx_1), \tag{5}$$

where

$$\Delta G(x_3, x_3 - Wx_1) := \tilde{G}(x_3) - \tilde{G}(x_3 - Wx_1).$$

By using the mean value theorem, one can write the above term as $D(t)Wx_1$ where $D(t)$ is a diagonal matrix made of derivative of the function g. By definition of h_m, every diagonal element of $D(t)$ belongs to the interval $[0, h_m]$.

One then rewrites System (3) as

$$\dot{x}_1 = -\frac{(I_N - D(t)W)x_1}{\tau_1} - \frac{1}{\tau_1}\tilde{G}(x_3) \tag{6a}$$

$$\dot{x}_2 = -\frac{x_2}{\tau_2} + \frac{x_1}{\tau_2} \tag{6b}$$

$$\dot{x}_3 = \frac{x_2}{\tau_3}. \tag{6c}$$

We have the following result, which provides a sufficient condition for the global asymptotic stability of the origin (6). It ensures in particular that the equilibrium (2) is unique and that all solutions of (1) converge to it. This result is slightly more general than what we actually need.

Proposition 1. *Consider System* (6)*, where* $D(\cdot)$ *is any measurable diagonal matrix-valued function defined for non negative times and taking values in* $[d_1, d_2]$ *with* $0 \le d_1 \le d_2 \le h_m$. *Assume moreover that the following two conditions holds:*

$$h_m\bar{\omega} < 1 \tag{7}$$

$$\tau_1 + \tau_2 > d\bar{\omega}\tau_2 + \frac{\tau_1\tau_2 h_m}{\tau_3(1-\bar{\omega}d_2)} + \delta\bar{\omega}\tau_2\sqrt{\frac{\tau_2 h_m}{\tau_3(1-\bar{\omega}d_2)}}, \tag{8}$$

where $d := \frac{d_1+d_2}{2}$, $\delta := \frac{d_2-d_1}{2}$. *Then the origin of* (6) *is globally asymptotically stable.*

For our purposes, we take $d_2 = h_m$ and $d_1 = 0$, yielding $d = \delta = \frac{h_m}{2}$.

Remark 1. Note, in the proof of the proposition, we will use systematically the following trivial fact. If M is an $N \times N$ real symmetric matrix and $x \in \mathbb{R}^N$, then $|Mx| \le \mu_M|x|$, where μ_M is the largest value over the $|\lambda|$'s, where λ is any eigenvalue of M.

Proof of Proposition 1: We first prove that the coordinate functions $x_1(\cdot)$ and $x_2(\cdot)$ are ultimately bounded, i.e., for any trajectory $x(\cdot)$ of System (6), there exists a time $T_{x(\cdot)}$ such that

$$\max\{|x_1(t)|;\ |x_2(t)|\} \le \frac{2G_m}{1-h_m d_2\bar{\omega}}, \quad \forall t \ge T_{x(\cdot)}. \tag{9}$$

To see that, notice that the time derivative of $\tau_1\frac{\|x_1\|^2}{2}$ along the trajectories of System (6) is equal to $\tau_1 x_1^T\dot{x}_1$. Consequently, by using Cauchy-Schwarz inequality and Remark 1,

$$\begin{aligned}\tau_1 x_1^T\dot{x}_1 &\le -|x_1|^2 + |D(t)x_1||Wx_1| + G_m|x_1| \\ &\le -(1-h_m d_2\bar{\omega})|x_1|\left(|x_1| - \frac{G_m}{1-h_m d_2\bar{\omega}}\right).\end{aligned}$$

In view of classical Lyapunov results (see e.g. [1]), the above implies Eq. (9) as far as $x_1(\cdot)$ is concerned. Similarly, the time derivative of $\tau_2 \frac{\|x_2\|^2}{2}$ along trajectories of System (6) is equal to $\tau_2 x_2^T \dot{x}_2$ and one has, by using Cauchy-Schwarz inequality and Remark 1,

$$\tau_2 x_2^T \dot{x}_2 \leq -|x_2|(|x_2| - |x_1|),$$

which establishes the $x_2(\cdot)$-part of (9).

Now, for each $i \in \{1, \dots, N\}$, define $k_i : \mathbb{R} \to \mathbb{R}^+$ as

$$k_i(q) := \int_0^q \tilde{G}_i(s) ds = \int_0^q \left(g(s - u_i^* + g^{-1}(-r_{goal})) + r_{goal} \right) ds, \quad \forall q \in \mathbb{R},$$

and the corresponding $K : \mathbb{R}^N \to \mathbb{R}^+$ as

$$K(z) := \sum_{i=1}^{N} k_i(z_i), \quad \forall z \in \mathbb{R}^N.$$

Let $m > 0$ be a constant to be fixed later. Consider the real-valued function V defined over $\mathbb{R}^{3N}$ by

$$\begin{aligned} V(x) :=\ & \tau_1 \tau_3 m \frac{|x_1|^2}{2} + \frac{\tau_2}{2} x_2^T \left((\tau_1 + \tau_2) I_N - \tau_2 d W \right) x_2 \\ & + \tau_3(\tau_3 m + \tau_2) K(x_3) + \tau_2 \tau_3 m \tilde{G}(x_3)^T x_2 + \tau_2 \tau_1 x_2^T x_1. \end{aligned} \tag{10}$$

In general, V is not bounded below over $\mathbb{R}^{3N}$ but, because the scalar number in front of $K(\cdot)$ is positive and recalling that $x_1(\cdot)$ and $x_2(\cdot)$ are ultimately bounded, it is immediate to see that $V(x(\cdot))$ is ultimately bounded by below along the trajectories of System (6). Moreover, a lengthy but easy computation yields that the time derivative of V along trajectories of System (6) is given by

$$\dot{V} = -(\tau_3 m - \tau_1)|x_1|^2 + \tau_3 m x_1^T D(t) W x_1 - x_2^T B x_2 + \tau_2 x_1^T D_1(t) W x_2,$$

where $B = (\tau_1 + \tau_2) I_N - \tau_2 d W - \tau_2 m \tilde{G}'(x_3(t))$ and $D_1(t) = D(t) - d I_N$. By using Cauchy-Schwarz inequality and Remark 1, the above equation becomes

$$\begin{aligned} \dot{V} \leq & - \left(\tau_3 m (1 - \bar{\omega} d) - \tau_1 \right) |x_1|^2 \\ & - \left((\tau_1 + \tau_2) - \tau_2 d \bar{\omega} - \tau_2 m h_m \right) |x_2|^2 + \delta \bar{\omega} \tau_2 |x_1||x_2|. \end{aligned} \tag{11}$$

We seek a condition on the data of the problem so that there exists $m > 0$ such that the right-hand side of Eq. (11) is a negative definite quadratic form in (x_1, x_2). This is equivalent to looking for a positive m such that

$$\alpha m - \tau_1 > 0, \ \beta - \tau_2 m h_m > 0, \ (\alpha m - \tau_1)(\beta - \tau_2 m h_m) > \gamma^2, \tag{12}$$

with $\alpha := \tau_3(1 - \bar{\omega} d_2)$, $\beta := \tau_1 + \tau_2 - \tau_2 d \bar{\omega}$ and $\gamma := \frac{\delta \bar{\omega} \tau_2}{2}$. The first two conditions are equivalent to the inequality $\frac{\beta}{\tau_2 h_m} > \frac{\tau_1}{\alpha}$, and after simplification, the overall condition reads

$$\frac{\beta}{\tau_2 h_m} - \frac{\tau_1}{\alpha} > \frac{2\gamma}{\sqrt{\alpha \tau_2 h_m}}, \tag{13}$$

which is Eq. (8). Under this condition, we can find a positive value of m such that all three conditions in 12 hold. Consequently, there exists two positive constants C_1, C_2 such that for every trajectory $x(\cdot)$ of System (6), there exists a time $T_{x(\cdot)}$ so that

$$\dot{V}(t) \leq -C_1 |x_1(t)|^2 - C_2 |x_2(t)|^2, \quad \forall t \geq T_{x(\cdot)}.$$

For $\varepsilon > 0$ to be fixed later, consider the real-valued function $\tilde{V}$ defined over $\mathbb{R}^{3N}$ by

$$\tilde{V}(x) := V(x) + \tau_1\tau_3\varepsilon\tilde{G}(x_3)^T x_1,$$

and notice that $\tilde{V}$ is ultimately bounded as V since $\tilde{G}$ is bounded. For $t \geq T_{x(\cdot)}$, the time derivative of $\tilde{V}$ along the trajectories of System (6) satisfies, after easy estimates,

$$\begin{aligned}\dot{\tilde{V}} &\leq -C_1|x_1|^2 - C_2|x_2|^2 + \varepsilon\tau_1 h_m|x_1||x_2| \\ &+\varepsilon\tau_3(1+d_2\bar{\omega})|\tilde{G}(x_3)||x_1| - \varepsilon\tau_3|\tilde{G}(x_3)|^2.\end{aligned}$$

By using the standard estimate $2|ab| \leq a^2 + b^2$ for any real numbers a, b, we deduce that, for $\varepsilon > 0$ small enough and for all $t \geq T_{x(\cdot)}$,

$$\dot{\tilde{V}} \leq -\frac{C_2}{2}|x_2|^2 - \frac{C_1}{2}|x_1|^2 + \varepsilon\tau_3(1+d_2\bar{\omega})|\tilde{G}(x_3)||x_1| - \varepsilon\tau_3|\tilde{G}(x_3)|^2.$$

The last three terms of the right-hand side of the above inequality clearly define a negative definite form in $(x_1, G(x_3))$ for $\varepsilon > 0$ small enough and, consequently, we can pick ε small enough that, for $t \geq T_{x(\cdot)}$,

$$\dot{\tilde{V}} \leq -\frac{C_1}{4}|x_1|^2 - \frac{C_2}{2}|x_2|^2 - \frac{\varepsilon\tau_3}{2}|\tilde{G}(x_3)|^2.$$

Integrating on both sides, and recalling that $\tilde{V}$ is ultimately bounded from below along the trajectories, it follows that the three integrals $\int_0^\infty |x_1(t)|^2 dt$, $\int_0^\infty |x_2(t)|^2 dt$ and $\int_0^\infty |\tilde{G}(x_3(t))|^2 dt$ are finite. Since the time derivatives of the functions $x_1(\cdot)$, $x_2(\cdot)$ and $\tilde{G}(x_3(\cdot))$ are bounded and recalling that $\tilde{G}(0) = 0$, we conclude from Barbalat's lemma [1] that the trajectory $x(\cdot)$ tends to zero as t tends to infinity.

2 Stability of single non-linear neuron with recurrence

Here we derive stability of the dominant mode of the full network. The model under concern is a 3D dimensional projection of the full model

$$\dot{r}_1 = \frac{1}{\tau_1}(-r_1 + g(u + wr_1 - r_3)) \quad (14a)$$

$$\dot{r}_2 = \frac{1}{\tau_2}(-r_2 + r_1) \quad (14b)$$

$$\dot{r}_3 = \frac{1}{\tau_3}(r_2 - r_{goal}). \quad (14c)$$

For a constant input $u = u^*$ and a given r_{goal}, we can compute the equilibrium r^* of this system by solving $\dot{r}_1 = \dot{r}_2 = \dot{r}_3 = 0$. This gives:

$$r_1^* = r_{goal} \quad (15a)$$

$$r_2^* = r_{goal} \quad (15b)$$

$$r_3^* = u^* + wr_{goal} - g^{-1}(r_{goal}). \quad (15c)$$

Let $x := r - r^*$, then the system can be equivalently written as

$$\dot{x} = Ax + B\sigma(Cx) \quad (16)$$

where $\sigma : \mathbb{R} \to \mathbb{R}$ is the continuous function defined as

$$\sigma(v) := g(v + r_3^* - u^*) - r_1^* = g\big(v + g^{-1}(r_{goal})\big) - r_{goal}, \quad \forall v \in \mathbb{R}, \quad (17)$$

and the matrices A, B and C are given by

$$A = \begin{pmatrix} -1/\tau_1 & 0 & 0 \\ 1/\tau_2 & -1/\tau_2 & 0 \\ 0 & 1/\tau_3 & 0 \end{pmatrix} \tag{18}$$

$$B = \begin{pmatrix} 1/\tau_1 \\ 0 \\ 0 \end{pmatrix} \tag{19}$$

$$C = \begin{pmatrix} w & 0 & -1 \end{pmatrix}. \tag{20}$$

We have the following result.

Proposition 2. *The solutions of the system (3)-(20) all converge to zero (or, equivalently, the solutions of (14) all converge to the equilibrium r^* given in (15)) for all continuous nonlinearities satisfying*

$$0 < \frac{\sigma(v)}{v} < L, \quad \forall v \in \mathbb{R}, \tag{21}$$

if and only if it holds that $Lw < 1$ and

$$\tau_3 \geq \frac{L}{1 - wL}\left(\frac{\tau_1 + \tau_2}{\tau_1 + (1 - wL)\tau_2}\right). \tag{22}$$

Eq. (21)-(22) constitutes a more general assumption as is does not require the differentiability of σ nor its monotonicity. In particular, it encompasses classical saturation functions: linear in a neighborhood of zero and equal to a constant out of this neighborhood. It also allows functions that tend to zero as $v \to \infty$.

Proof. Proposition 2 is an immediate consequence of Aizerman's conjecture, which we recall below for the sake of completeness.

Aizerman's conjecture (1949): The system (3) globally asymptotically stable for all nonlinear functions satisfying

$$0 < \frac{\sigma(v)}{v} < \ell, \quad \forall v \in \mathbb{R}$$

if and only if, given any $k \in (0; \ell)$, the eigenvalues of the matrix $A + kBC$ all have negative real parts.

Aizerman's conjecture has been disproved in general for systems of dimension greater than 3 [2, 3]. Nonetheless, it happens to hold true for the particular system (14). More precisely, we recall the following statement from [4]. □

Lemma 1. *[4] Let $A \in \mathbb{R}^{3\times3}$ be a matrix having no eigenvalue with positive real part. Let $B \in \mathbb{R}^{3\times1}$ and $C \in \mathbb{R}^{1\times3}$ be such that (A, B) is controllable and (A, C) is observable. If the transfer function $G(s) := C(sI - A)^{-1}B$ can be written as*

$$G(s) = \frac{\beta_0 s^2 + \beta_1 s + \beta_2}{s(s^2 + \alpha_1 s + \alpha_0)}$$

for some $\alpha_i, \beta_i \in \mathbb{R}$, $i \in \{1, 2\}$, then Aizerman's conjecture holds true for the system $\dot{x} = Ax + B\sigma(Cx)$.

Let us apply Lemma 1 to system (14). Since the matrices[1] $[B\ \ AB\ \ A^2B]$ and $[C\ \ CA\ \ CA^2]^T$ both have full rank, the pairs (A, B) and (A, C) are respectively controllable and observable. Moreover the transfer function $G(s)$ introduced in the statement of Lemma 1 reads in our case:

[1] respectively called Kalman's controllability and observability matrices in the contol theory literature

$$G(s) = \frac{1}{\tau_1\tau_2\tau_3}\frac{-1 + s\tau_3 w + s^2\tau_2\tau_3 w}{s\left(s^2 + \frac{\tau_1+\tau_2}{\tau_1\tau_2}s + \frac{1}{\tau_1\tau_2}\right)}.$$

Thus, all the assumptions of Lemma 1 are satisfied. So the system (3) is globally asymptotically stable (in particular, all solutions of (3) converge to the origin) for all nonlinearities σ satisfying (21) if and only if, given any $k \in (0; L)$, all the eigenvalues of the matrix $A_k := A + kBC$ have negative real parts. Here, A_k reads:

$$A_k := \begin{pmatrix} (-1+kw)/\tau_1 & 0 & -k/\tau_1 \\ 1/\tau_2 & -1/\tau_2 & 0 \\ 0 & 1/\tau_3 & 0 \end{pmatrix}.$$

Consequently, its characteristic polynomial is

$$\det(\lambda I - A_k) = \lambda^3 + \left(\frac{1-kw}{\tau_1} + \frac{1}{\tau_2}\right)\lambda^2 + \frac{(1-kw)}{\tau_1\tau_2}\lambda + \frac{k}{\tau_1\tau_2\tau_3}.$$

Routh's criterion[2] then provides the following necessary and sufficient conditions for the roots of this polynomial (i.e. the eigenvalues of A_k) to all have negative real part: $k > 0$, $1 - kw > 0$, and $k < \tau_3(1-kw)(\frac{1-kw}{\tau_1} + 1/\tau_2)$: we recover the condition (22), which concludes the proof.

References

[1] H. K. Khalil, Nonlinear systems, 3rd Edition, Prentice hall Upper Saddle River, 2002.

[2] R. Fitts, Two counterexamples to Aizerman's conjecture, Automatic Control, IEEE Transactions on 11 (3) (1966) 553–556.

[3] V. Bragin, V. Vagaitsev, N. Kuznetsov, G. Leonov, Algorithms for finding hidden oscillations in nonlinear systems. the Aizerman and Kalman conjectures and Chua's circuits, Journal of Computer and Systems Sciences International 50 (4) (2011) 511–543.
URL http://link.springer.com/article/10.1134/S106423071104006X

[4] K. Fujii, K. Shoji, Verification of the Aizerman and/or Kalman conjecture, IEEE Transactions on Automatic Control 17 (3) (1972) 406–408.

[2] Routh's criterion is also a classical tool in control theory and matrix theory. It is a simple way to test the stability of a polynomial based on a table involving its coefficients.
